# A comparative study of the genetically improved Abbassa Nile tilapia strain (GIANT-G9) and a commercial strain in Egypt: growth vs. stress response

**DOI:** 10.1007/s11259-024-10639-y

**Published:** 2025-01-14

**Authors:** Eman Ahmed Bedir, Mohamed Mohamed Said, Rasha A. Al Wakeel, Ahmed M. Nasr-Allah, Haitham G. Abo-Al-Ela

**Affiliations:** 1 Department of Aquaculture, Faculty of Fish Resources, Suez University, Suez, 43221 Egypt; 2 Department of Physiology, Faculty of Veterinary Medicine, Kafrelsheikh University, Kafrelsheikh, 33516 Egypt; 3 WorldFish, Abbassa, Abu Hammad, Sharkia, Egypt; 4 Genetics and Biotechnology, Department of Aquaculture, Faculty of Fish Resources, Suez University, Suez, 43221 Egypt

**Keywords:** Abbassa strain, Ammonia stress, Growth, Nile tilapia, Temperature stress

## Abstract

Selective breeding is a potent method for developing strains with enhanced traits. This study compared the growth performance and stress responses of the genetically improved Abbassa Nile tilapia strain (G9; GIANT-G9) with a local commercial strain over 12 weeks, followed by exposure to stressors including high ammonia (10 mg TAN/L), elevated temperature (37 °C), and both for three days. The GIANT-G9 showed superior growth, including greater weight gain, final weight, length gain, specific growth rate, and protein efficiency ratio, as well as a lower feed conversion ratio and condition factor compared to the commercial strain. The expression of growth hormone in the brain of the GIANT‐G9 increased significantly after 6 weeks, although it slightly decreased after 12 weeks. Growth hormone receptor 1 expression also increased significantly after 6 weeks. Muscle insulin-like growth factors (*igf1* and *igf2*) levels up-regulated significantly only after 12 weeks in the GIANT‐G9. Under stress, serum enzymes (alanine aminotransferase, aspartate aminotransferase, and alkaline phosphatase (ALP)) were significantly higher in the GIANT‐G9, while the commercial strain had lower levels. No significant changes were observed in liver ALP activity among stressed strains. Under stress, the GIANT‐G9 exhibited marked upregulation of splenic Toll-like receptors (*tlr2*, *tlr9*, *tlr21*), myeloid differentiation primary response protein 88 (*myd88*), nuclear factor kappa B (*nf-κB*), interleukin (*il*) *1β*, and *il6*. Notably, *il6* expression was higher than *il1β* in the spleen, with the opposite pattern in the head kidney. In response to immune stimulation, globulin levels significantly increased in the GIANT‐G9 but with similar values to the stressed commercial strain. Myostatin expression increased in the spleen of the stressed GIANT‐G9. The commercial strain exhibited the best liver catalase and superoxide dismutase activities under stress, while the GIANT‐G9 showed increased liver glutathione-*S*-transferase (GST) activity after exposure to ammonia and temperature stress. Serum lysozyme activity increased in the stressed commercial strain and under temperature stress in the GIANT‐G9 but decreased under other stress conditions. Overall, the stressed commercial strain demonstrated higher survivability than the stressed GIANT‐G9. The study revealed significant interactions between strains and stress factors. The GIANT‐G9 exhibited higher growth rates but lower antioxidant and immune capacities compared to the commercial strain at the juvenile stage of life and production cycle.

## Introduction

Tilapia (omnivorous cichlids) could be likened to aquatic chickens for their rapid and efficient growth. They possess a sweet and subtle flavour and are remarkably easy to cultivate. As one of the fastest-growing aquaculture products, tilapias may very well represent the future of the industry. Among the various tilapia species, the Nile tilapia (*Oreochromis niloticus*) has swiftly ascended the ranks of farmed fish (Cressey 2009). Following carp, tilapias stand as the second most popular farmed fish globally (Eknath and Hulata 2009; Yacout et al. 2016). The initial cultivation of tilapias took place in Kenya in 1924 (*O. spilurus niger*) and in Zaire in 1937 (*Tilapia rendalli* and *O. nyasalapia macrochir*) (Chimits 1957). According to FAO data, Egypt is one of the world’s leading producers of tilapia, with production expected to continue increasing in the future. From 2022 to 2023, both China and Egypt achieved a 5% growth in tilapia production (Darryl 2023; FAO 2022). Decades of research, both past and ongoing, aim to further enhance the productivity and health of Nile tilapia (Atef et al. 2024; Obirikorang et al. 2019; Ridha and Cruz 2003).

Tilapias boast numerous advantages over other cultured species, including their rapid growth and resistance to disease. Generally, some cultured species can tolerate concentrations of 0.1 mg/L of dissolved oxygen and 2.4 mg/L of unionized ammonia (Lovell 1998). While they originate from freshwater environments, tilapias are euryhaline, meaning they can thrive in and adapt well to saline water. They, however, cannot survive temperatures significantly below 12 °C (Lovell 1998).

Cultured fish face various stressors, including extreme temperatures, ammonia, heavy metal, and xenobiotic pollution, rendering them susceptible to diseases and reduced productivity. These factors can lead to an overproduction of reactive oxygen species (ROS), surpassing the body’s defensive antioxidant system, resulting in stress and disruptions to bodily functions (Abo-Al-Ela and Faggio 2021; Hamed et al. 2024).

ROS are endogenous, highly reactive, oxygen-bearing molecules that include two species: free radicals such as superoxide radical anion (O2˙^−^) and hydroxyl radicals (OH˙) and non-radicals such as hydrogen peroxide (H_2_O_2_), ozone (O_3_) and singlet oxygen (^1^O_2_) (Krumova and Cosa 2016). ROS or related reactive species can act as first messengers, in which ROS levels are transiently increasing, fulfill a signaling role that leads to physiological responses, or as second messengers, where ROS levels cause redox modulation, leading to physiological or pathophysiological responses and subsequent cell injury (Li et al. 2016). The latter potential pathway alters the expression of cellular products, such as inflammatory mediators and adhesion molecules (Li et al. 2016; Wu et al. 2017).

Animal breeding and selection serve as valuable tools for enhancing desired traits in animals. However, the process is time-consuming and exhibits gradual progress (Rezk et al. 2009; Thodesen et al. 2012). Ongoing research aims to refine the selection process, thereby improving the outcomes of selective breeding (de Verdal et al. 2022; Humanes et al. 2024). In Egypt, the selective breeding of Nile tilapia has demonstrated sustainable increases in harvest weight, with recorded gains of 6.64% and 6.96% over two consecutive years (Rezk et al. 2009). Similarly, in China, a six-generation multi-trait selection of the genetically improved farmed tilapia (GIFT breed) by the WorldFish Center resulted in significant growth improvements. This effort manifested in remarkable increases of 60–90% in body weights at harvest (Thodesen et al. 2011). In 2001, WorldFish Egypt initiated a selective breeding program to develop the genetically improved Abbassa Nile tilapia (GIANT) strain, utilizing the same technology used by Rezk et al. (2009) to produce a strain with enhanced growth rates and higher survival (Ibrahim et al. 2019).

It is noteworthy that the GIANT strain in Egypt and the GIFT strain in the Philippines, when reared in two distinct input environments, did not exhibit significant changes in family rankings, indicating a low genotype-environment interaction (Eknath et al. 2007; Khaw et al. 2009). This suggests that these strains may perform consistently across different environmental conditions, though it does not imply resilience of the production systems themselves to environmental variations (Eknath et al. 2007; Khaw et al. 2009).

Selective breeding involves the transmission of the desirable gene-associated traits from one generation to another, encompassing the genetic composition that, in turn, modulates the expression of genes and phenotype (Robinson et al. 2008; Tan et al. 2020; Yáñez et al. 2020). In aquaculture, genome-wide association studies have revealed that productive traits are often polygenic, influenced by multiple loci (Houston et al. 2020). Consequently, it is crucial to delve into the molecular events beyond the improvements resulting from selective breeding to comprehend the potential interaction between genetic elements and the key modulators of these traits.

This study aims to examine differences in growth performance and stress responses—specifically to elevated temperature and ammonia—between two distinct Nile tilapia strains: the 9th generation genetically improved Abbassa Nile tilapia (GIANT-G9) strain and a commercial local strain. The investigation focuses on growth performance, expression of growth- and immune-related genes, and potential changes in biochemical parameters.

## Materials and methods

### Fish rearing and management

All-male Nile tilapia were obtained from two distinct sources. A commercial local strain was obtained from a farm in West El-Qantara, Ismailia, Egypt, which distributes its production widely within the local market. The GIANT-G9 strain (commercially available) was acquired from WorldFish in Abbassa, Abou-Hammad, Sharkia, Egypt. The fish weights were 6.01 ± 0.08 g and 6.10 ± 0.09 g for the GIANT-G9 strain and the commercial local strain, respectively. They were hand-fed three times daily with a powdered fresh feed containing 30% crude protein, 5.70% crude fat, 7.10% ash, 90.35% dry matter, and 46.55% nitrogen-free extract (Grand Aqua^®^, Egypt).

**Fig. 1:**
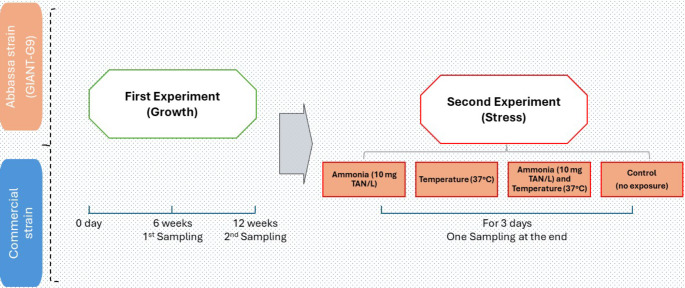
Diagrammatic illustration of the experimental plan. TAN, total ammonium nitrogen

The study was divided into two distinct experiments, as illustrated in Fig. 1. The first experiment focused on comparing the growth of two Nile tilapia strains, while the second experiment aimed to assess differences in stress responses between the strains.

In the first experiment, a portion of the fish was stocked in 100-liter round plastic tanks, with four tanks designated for each strain and 20 fingerlings per tank. Before the experiment began, the fish were allowed a two-week acclimation period. The first experiment lasted for 84 days (12 weeks). For the first six weeks, the feeding rate was set at 5% of their total body weight per day, and for the remaining six weeks, it was reduced to 4%.

Throughout both experiments, continuous aeration and daily exchanges of fresh, dechlorinated tap water were provided to maintain water quality. Key water parameters were monitored regularly to ensure optimal conditions: dissolved oxygen (DO) and temperature were measured with a HANNA portable DO meter, pH levels were recorded using a HANNA portable pH meter, and ammonia levels were assessed with a HANNA medium-range ammonia reagent kit.

During the first part of the experiment, the water temperature, dissolved oxygen, pH, and ammonia levels remained within normal ranges, as shown in Table 1. During the second part of the experiment, which involved stress exposure, these parameters changed according to the type of stress, as detailed in Table 2.

**Table 1:** Water quality criteria of the first experiment (growth)

Starin	Water temperature (°C)	DO (mg/L)	pH	Ammonia (mg/L)
GIANT-G9	28.60 ± 0.000	5.870 ± 0.034	7.598 ± 0.043	0.0723 ± 0.006
Commercial	28.54 ± 0.000	6.033 ± 0.080	7.885 ± 0.019	0.0825 ± 0.009

The data expressed as mean ± SD

**Table 2:** Water quality criteria in the second experiment (stress)

Strain	Treatment	Water temperature (°C)	DO (mg/L)	pH	Ammonia (mg/L)
GIANT-G9	Control	28.667 ± 1.258	7.133 ± 0.321	8.033 ± 0.503	0.093 ± 0.137
	Ammonia	29.167 ± 1.040	7.033 ± 0.208	8.033 ± 0.115	0.567 ± 2.466
	Temperature	35.833 ± 1.607	7.067 ± 0.351	8.233 ± 0.252	0.157 ± 0.150
	Ammonia and Temperature	36.667 ± 0.577	6.933 ± 0.513	8.067 ± 0.208	0.567 ± 1.443
Commercial	Control	28.167 ± 1.040	6.900 ± 0.361	7.833 ± 0.305	0.077 ± 0.124
	Ammonia	28.833 ± 0.289	6.867 ± 0.231	8.010 ± 0.100	0.500 ± 2.598
	Temperature	35.667 ± 1.258	6.967 ± 0.208	8.000 ± 0.200	0.123 ± 0.146
	Ammonia and Temperature	36.333 ± 1.040	6.733 ± 0.252	8.033 ± 0.252	0.533 ± 1.607

The data expressed as mean ± SD

Dead fish were regularly checked, and their survivability was determined as a percentage at the end of each experimental period.

### Growth performance, feed utilization parameters, and survivability

The formulas for various growth and performance metrics are defined as follows:

- Specific growth rate (SGR % day^–1^)) = (ln W*t*– ln W*o*) / *t* × 100, where ln represents the natural logarithm, W*t* is the final weight, W*o* is the initial weight, and *t* is the rearing duration in days.
- Weight gain (WG; g) = W*t*– W*o*, where W*t* is the final weight, W*o* is the initial weight.
- Length gain (LG; cm) = L*t*– L*o*, where L*t* is the final length, and L*o* is the initial length.
- Feed conversion ratio (FCR) = Feed intake (FI; g) / Weight gain (WG; g).
- Protein efficiency ratio (PER) = Wet weight gain (g) / Total protein intake (g).
- Condition factor (CF) (%) = Total weight / Standard length^3^.
- Hepato-somatic index (HIS) (%) = (Liver weight / Total body weight) × 100.

### Stress exposure

At the end of the growth experiment, fish from each strain were divided into four experimental groups. The second experiment involved a total of 240 fish per strain, allocated into 12 tanks for each strain, with three replicates per treatment group. Following the first experiment, the fish acclimated for seven days. Each group was subjected to a different treatment: a control (no exposure), temperature stress at 37 °C, ammonia stress at 10 mg/L total ammonium nitrogen (TAN), or a combination of elevated temperature and high ammonia under the same conditions. This exposure experiment lasted for three days, during which the fish were fed the same commercial feed used throughout the experiment. The feed was administered at a rate of 3% of the fish’s body weight, twice daily at 08:00 am and 2:00 pm.

For the temperature stress, the temperature was increased by 2 °C every 2 h until reaching 37 °C. For the high ammonia concentration stress, an ammonium chloride (NH_4_Cl; Fisher Scientific, USA) solution was prepared to achieve 10 mg TAN/L, following the protocols of Hegazi et al. (2010) and Benli et al. (2008). Water quality maintenance procedures were consistently followed, including the use of freshly prepared ammonium chloride solution to maintain TAN at 10 mg/L and pre-warmed water at 37 °C. These measures ensured that the stress was solely induced by ammonia and/or temperature.

### Sampling

For RNA extraction and gene expression analysis, samples were collected from both the brain and muscle at two time points: after six weeks and at the completion of the 12-week growth experiment. Three samples per replicate were taken at each time point. At the end of the second experiment, tissue samples (*n* = 5 from each tank) were taken from the head kidney and spleen. These samples were promptly frozen and stored at − 80ºC. Additionally, serum samples were collected for biochemical analysis, and liver samples were taken for the analysis of antioxidant parameters (*n* = 5 from each tank).

### Gene expression analysis

RNA extraction was carried out using TRIzol reagent (Invitrogen™ Life Technologies, US). The extracted RNA’s quality and quantity were assessed using a nanodrop (NanoPhotometer^®^ NP80, Implen, Germany) and by running it through 1% agarose gel electrophoresis. The synthesis of cDNA was performed using 2 µg of RNA and kits from Bioline, based in London, UK.

Real-time PCR was conducted using specific primers targeting growth hormone (*gh*), growth hormone receptor 1 (*ghr1*), insulin-like growth factor (*igf*) 1 and 2, Toll-like receptors (*tlr*) 2, 9, and 21, myeloid differentiation primary response protein 88 (*myd88*), nuclear factor kappa B (*nf-κB*), interleukin (*il*) 1β and 6, and myostatin (*mstn*), along with the reference genes elongation factor 1 alpha (*ef1α*) and ubiquitin-conjugating enzyme (*ubce*) (Table 3). The cycling parameters for the real-time PCR were set as follows: 95 °C for 3 min, followed by 40 cycles of 95 °C for 15 s, 60 °C for 1 min, and 72 °C for 30 s. The specificity of the PCR products was confirmed through dissociation analysis. *Igf1* and *Igf2* were analyzed in the muscle; *gh* and *ghr1* were analyzed in the brain; *tlr2*, *tlr9*, *tlr21*, *myd88*, *nf-κB*, *il1β*, *il6*, and *mstn* were analyzed in the spleen; and *nf-κB*, *il1β*, and *il6* were analyzed in the head kidney.

**Table 3:** Primers used for real-time PCR

Gene name	forward (sense, left) primer (5′−3′)	reverse (antisense, right) primer (5′−3′)	amplicon size (bp)	GenBank accession no.	References
*gh*	CTGTCTGTCTGTCTGTCAGTCGT	AGAGGAGACGCCCAAACAC	60	M26916; M84774	Ber and Daniel (1992) and Rentier-Delrue et al. (1989)
*ghr1*	TCTCAGCAGAACCGATTAATGA	TTTGATTTTGGGTGCAGGA	60	EF052861	Herkenhoff et al. (2020)
*igf1*	CCCGAACTTCCTCGACTTGA	CCTCAGCCAGACAAGACAAAAA	101	EU272149	
*igf2*	CCCCTGATCAGCCTTCCTA	GACAAAGTTGTCCGTGGTGA	60	EU272150	
*tlr2*	TGTCATGTGCCATCAGGTTT	TCTGCCTTATCTGTGCGTTG	180	JQ809459.1; XM_013264298.3	The current study
*tlr9*	CAGTTTTCGTGCTGTCCAGA	TGCACAATCGTTTTCTCAGC	174	MT338570.1; XM_031753215.2	The current study
*tlr21*	AACGGACTCACCGTTTTACCA	GGAGAAGTTCTGAATGCCCAT	243	KJ010824.1; NM_001311317.1	Pang et al. (2017)
*myd88*	TAAACGGATGGTGGTGGTGA	GCGTTTACTTCGAGCTCCAG	106	KJ130039	Trung and Lee (2020)
*nf-κB*	CGACCACTACCTACACGCTC	GATGTCGTTTGAGGCATCGC	96	XM_019363515.2	Yu et al. (2022)
*il1β*	TGCACTGTCACTGACAGCCAA	ATGTTCAGGTGCACTATGCGG	113	DQ061114.1	Choi et al. (2007)
*il6*	ACAGAGGAGGCGGAGATG	GCAGTGCTTCGGGATAGAG	165	XM_019350387	Wei et al. (2018)
*mstn*	CACTGTGGACTTCGAGGACT	CTCTGGGGTTGGCTTTGTTC	143	KT987208.1	The current study
*ubce*	CTCTCAAATCAATGCCACTTCC	CCCTGGTGGAGGTTCCTTGT	130	XM_003460024	Yang et al. (2013)
*ef1α*	GCACGCTCTGCTGGCCTTT	GCGCTCAATCTTCCATCCC	250	AB075952	

*gh*, growth hormone; *ghr1*, growth hormone receptor 1; *igf*, insulin-like growth factor; *tlr*, Toll-like receptor; *myd88*, myeloid differentiation primary response protein 88; *nf-κB*, nuclear factor kappa B; *il*, interleukin; *mstn*, myostatin; *ubce*, ubiquitin-conjugating enzyme; *ef1α*, elongation factor 1 alpha. The primers designed in this study were generated using Primer3web (https://bioinfo.ut.ee/primer3/) and subsequently underwent specificity testing using the BLAST™ tool for primers (https://www.ncbi.nlm.nih.gov/tools/primer-blast/)

Gene expression was normalized using the geometric mean of the two reference genes *ef1α* and *ubce* as described in Chervoneva et al. (2010) and Atef et al. (2024). The expression data for *gh*, *ghr1*, and *igf1* and *igf2* were presented as 2^–ΔCt^ (Petakh et al. 2024; Rochman et al. 2014). However, the gene expression results of the second experiment were calculated using the Pfaffl (2001) method.

### Biochemical analysis of sera and liver samples

The serum lysozyme activity was measured using a fish lysozyme ELISA kit (Sunlong Biotech Co., China) at a wavelength of 450 nm, following the manufacturer’s instructions. Serum biochemical parameters were determined colorimetrically using ready-made chemical kits, according to the manufacturers’ instructions: alkaline phosphatase at 510 nm, alanine aminotransferase (ALT), aspartate aminotransferase (AST), and albumin (Biodiagnostic Co., Egypt), and total serum proteins (Spectrum Co., Egypt). Globulin content was calculated using the values of total proteins and albumin.

Liver homogenates were prepared using phosphate-buffered saline for the determination of superoxide dismutase (SOD) at 560 nm, catalase, and alkaline phosphatase (ALP) at 510 nm, and glutathione-*S*-transferase (GST) at 340 nm, following the kit instructions from Biodiagnostic Co., Egypt.

### Statistical analysis

The data were analyzed using IBM SPSS version 20. In the first experiment (growth), a t-test was employed to evaluate growth performance, feed utilization, carcass composition, and gene expression, with the tank mean as the data point. In the second experiment (stress), individual data points were used. A two-way ANOVA was conducted on the stress data to assess the interaction effect between strain and stress factor. Additionally, a one-way ANOVA followed by Tukey’s multiple comparison test was used to examine the results within the same strain. GraphPad Prism 9 (La Jolla, California, USA) was used for statistical analysis. Data are expressed as mean ± SD, with significance tested at the 0.05 level.

## Results

### Growth performance and feed utilization parameters

**Fig. 2:**
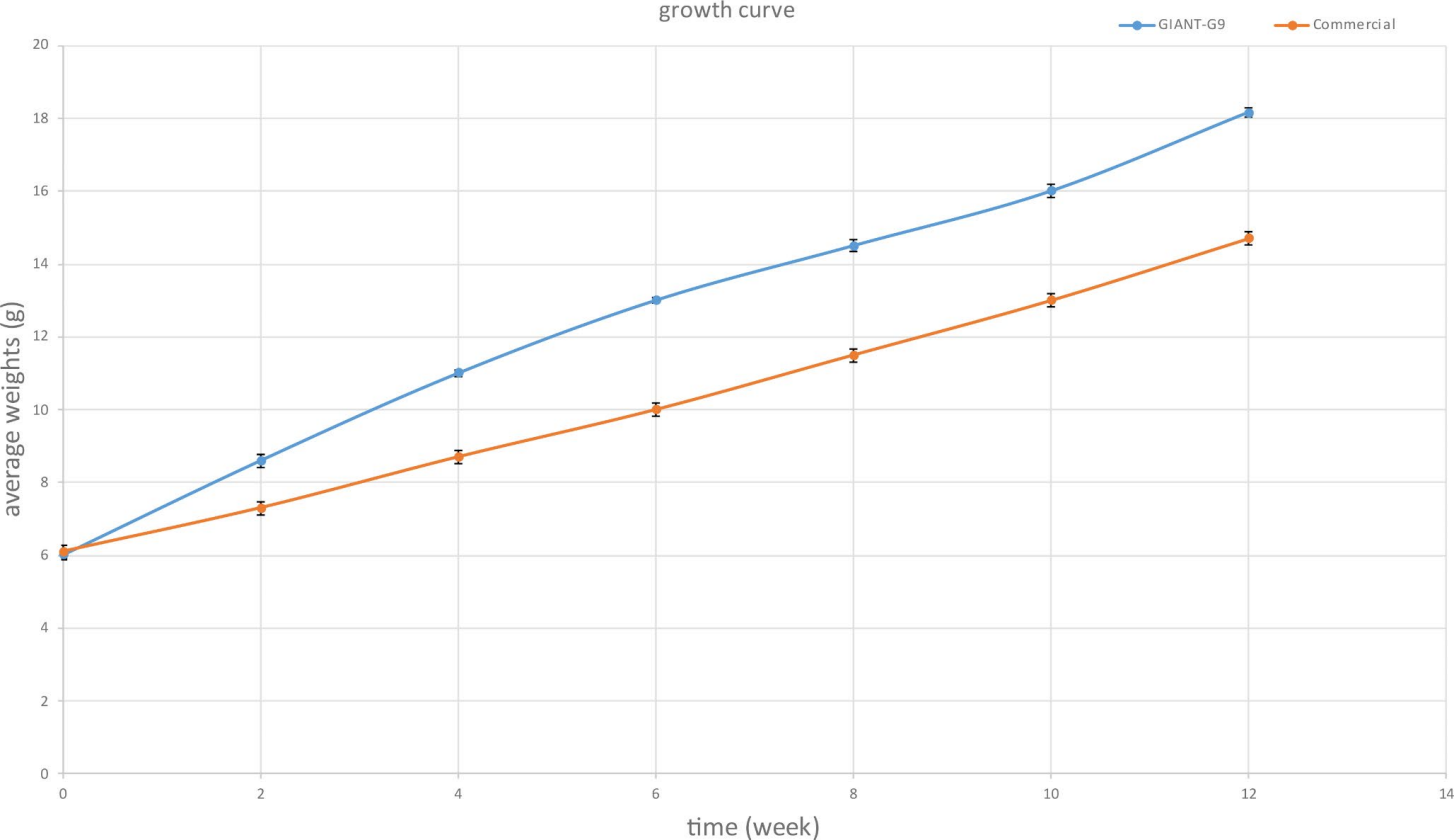
Average weights for two Nile tilapia strains (commercial strain and GIANT-G9), recorded every two weeks during the first experimental period (84 days; 12 weeks)

The GIANT-G9 exhibited consistent marked growth throughout the experiment, with a significant WG increase by 3.35 g compared to the commercial strain (Fig. 2). This growth was reflected in the fish’s FW and LG, with increases of 3.46 g and 3.03 cm, respectively, compared to the commercial strain (Table 4). The SGR was higher, while the FCR was lower in the GIANT‐G9 compared to the commercial strain. Additionally, the GIANT‐G9 had a higher PER and a lower CF % than the commercial strain (Table 4). FI and HIS did not differ significantly between the two strains.

**Table 4:** Growth performance of two Nile tilapia strains (commercial strain and GIANT-G9) reared for 84 days (12 weeks)

Growth parameters	GIANT-G9	Commercial strain	*P* value
IW (g)	6.01 ± 0.08	6.10 ± 0.09	0.193
WG (g)	12.08 ± 0.22	8.725 ± 0.26	< 0.0001
FW (g)	18.16 ± 0.32	14.70 ± 0.34	< 0.0001
LG (cm)	9.03 ± 0.05	6.00 ± 0.14	< 0.0001
FI (g/tank)	454.36 ± 52.73	461.168 ± 53.509	0.756
SGR (%)	0.57 ± 0.01	0.46 ± 0.01	< 0.0001
FCR	1.65 ± 0.05	2.30 ± 0.08	< 0.0001
PER	1.95 ± 0.06	1.40 ± 0.08	< 0.0001
CF (%)	0.54 ± 0.01	0.89 ± 0.02	< 0.0001
HIS (%)	0.88 ± 0.46	0.91 ± 0.61	0.930

IW, initial weight; WG, weight gain; FW, final weight; LG, length gain; FI, feed intake; SGR, specific growth rate; FCR, feed conversion ratio; PER, protein efficiency ratio; CF, condition factor; HIS, hepato-somatic index. The data expressed as mean ± SD

### Expression of growth-related genes

**Fig. 3:**
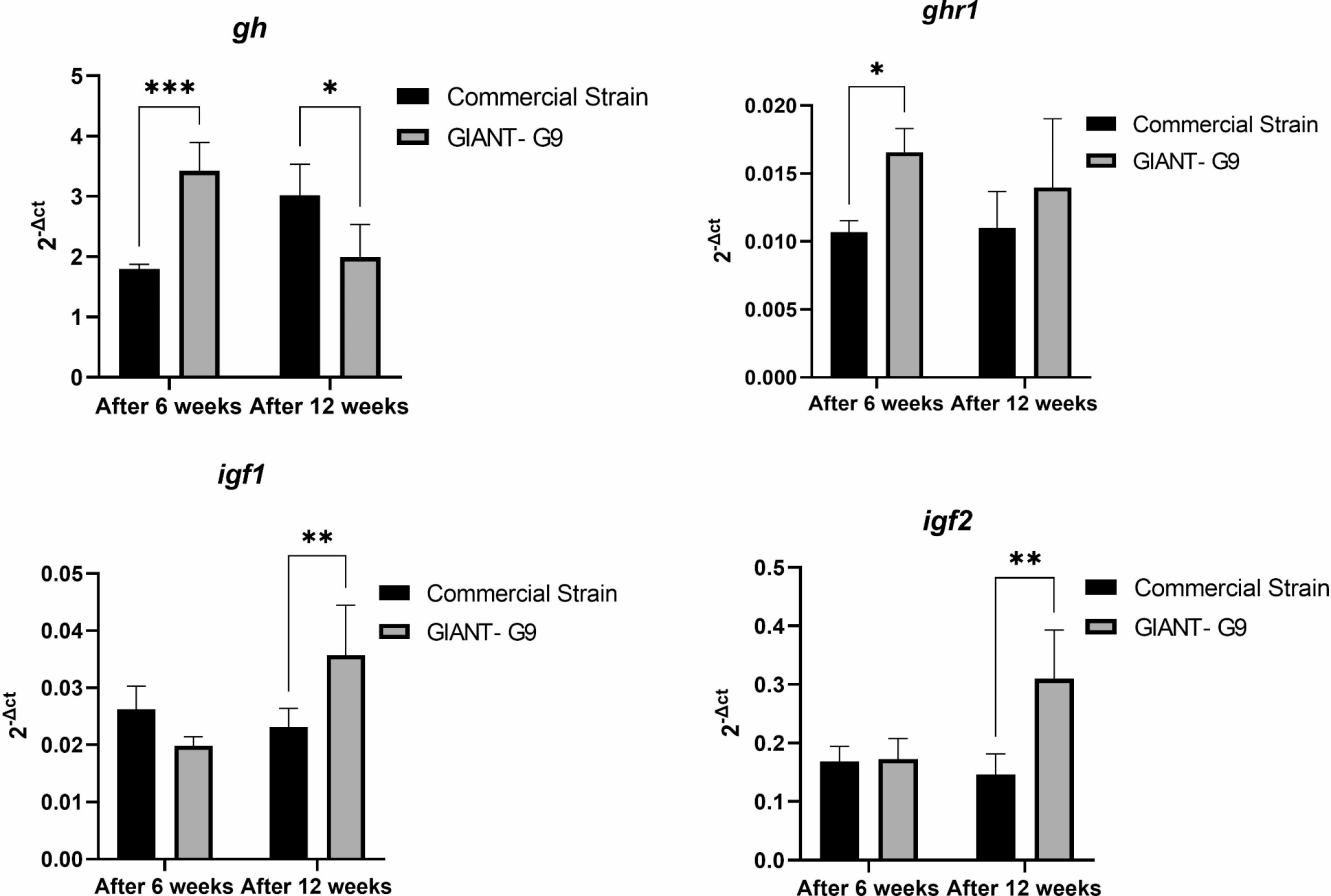
Gene expression results of growth hormone (*gh*) and growth hormone receptor 1 (*ghr1*) in the brain, and insulin-like growth factors 1 and 2 (*igf1* and *igf2*) in the muscle of two Nile tilapia strains (a commercial strain and the GIANT-G9) after 45 days and 90 days during the first experimental period (84 days; 12 weeks). The expression levels were normalized using the geometric mean of two reference genes, *ef1α* and *ubce*, and are presented as 2^–ΔCt^. Data are expressed as mean ± SD

In the GIANT-G9 strain, brain *gh* expression was significantly higher than in the commercial strain after 6 weeks, but showed a slight decrease after 12 weeks (Fig. 3). The expression of *ghr1* in the brain of the GIANT-G9 was significantly increased after 6 weeks, with nonsignificant increases after 12 weeks (Fig. 3). However, the expression of muscle *igf1* and *igf2* was significantly increased only after 12 weeks in the GIANT‐G9 compared to the commercial strain (Fig. 3).

### Survivability

No fish deaths were recorded during the first experiment (growth). However, in the second experiment (stress), the survivability rates for the GIANT-G9 were 95% for the control group (no stress), 75% for the ammonia stress group, 85% for the temperature stress group, and 40% for the group exposed to both ammonia and temperature stress. For the commercial strain, the survivability rates were 95% for the control group (no stress), 85% for the ammonia stress group, 90% for the temperature stress group, and 60% for the group exposed to both ammonia and temperature stress.

### Serum and liver biochemical parameters

Ammonia stress significantly increased the serum levels of ALT and AST in both strains and among other stress groups (Table 5). However, there was no significant difference between ammonia and temperature stress in the GIANT-G9. Notably, ALT and AST levels were significantly higher in the GIANT‐G9 compared to the commercial strain. Interestingly, exposure to both ammonia and temperature stressors decreased the serum levels of ALT and AST, but these changes were not significantly different from the control of each strain. The serum level of ALP increased in response to ammonia and decreased in response to temperature in the commercial strain (Table 5). In contrast, in the GIANT‐G9, ALP increased after exposure to ammonia and both stressors, with these increases being more pronounced than those observed in the commercial strain.

**Table 5:** Biochemical changes in the blood of two Nile tilapia strains (commercial strain and GIANT-G9) exposed to different stressors (ammonia at 10 mg/L TAN/L, temperature at 37ºC, both stressors combined) and a control group (no stress)

Parameters	Commercial Strain				GIANT-G9				*P* value
	Control	Ammonia	Temperature	Ammonia & Temperature	Control	Ammonia	Temperature	Ammonia & Temperature	Strain ⨉ Stress factor
ALT (U/L)	22.87 ± 0.37^a^	39.00 ± 0.58^c, **^	24.00 ± 0.24^a^	15.23 ± 0.19^d^	19.57 ± 0.55^ab^	48.00 ± 1.73^c, **^	19.93 ± 0.18^abc^	15.47 ± 0.32^a^	0.0010
AST (U/L)	25.39 ± 0.20^ab^	41.27 ± 1.18^c^	29.94 ± 0.08^b, *^	20.40 ± 0.31^a, **^	25.71 ± 0.19^a^	40.92 ± 0.74^b^	37.27 ± 0.86^b, *^	29.54 ± 0.72^a, **^	0.005
ALP (U/L)	20.20 ± 0.25^a^	29.36 ± 0.48^c, ****^	21.32 ± 0.42^a, *^	17.10 ± 0.15^d, ****^	20.23 ± 0.23^a^	39.71 ± 0.31^b, ****^	19.18 ± 0.43^a, *^	39.75 ± 0.29^b, ****^	< 0.0001
Total protein (g/dL)	6.48 ± 0.20^abc, **^	5.96 ± 0.09^a^	6.68 ± 0.12^a, b, c, *^	6.30 ± 0.16^a, b^	4.54 ± 0.18^a, **^	6.18 ± 0.15^ab^	5.08 ± 0.06^abc, *^	5.49 ± 0.22^bc^	0.009
Albumin (g/dL)	3.73 ± 0.25^abc^	3.08 ± 0.04^a^	3.71 ± 0.07^abc^	3.71 ± 0.23^ab, *^	3.13 ± 0.07^abc^	2.43 ± 0.26^a^	2.94 ± 0.10^abc^	2.52 ± 0.24^ab, *^	0.47
Globulin (g/dL)	2.75 ± 0.09^ab, ****^	2.87 ± 0.07^abc, ***^	2.97 ± 0.09^bc, ***^	2.58 ± 0.08^a, *^	1.40 ± 0.18^a, ****^	3.75 ± 0.12^b, ***^	2.14 ± 0.04^c, ***^	2.97 ± 0.07^d, *^	< 0.0001
Lysozyme activity (µg/mL)	4.41 ± 0.37^c^	7.59 ± 0.66^ab, ***^	7.07 ± 0.81^a^	7.74 ± 0.32 ^ab, ***^	4.91 ± 0.11^d^	2.14 ± 0.21^a, ***^	7.32 ± 0.87^c^	2.59 ± 0.15^a, ***^	0.0001

ALT, alanine aminotransferase, AST; aspartate aminotransferase; ALP, alkaline phosphatase. Values sharing no common lowercase letters are significantly different within the same strain, while asterisks denote significance within the same stress factor across different strains. ^*^ = significant difference at *p* < 0.05, ^**^ = significant difference at *p* < 0.01, ^***^ = significant difference at *p* < 0.001. The data expressed as mean ± SD

Changes in serum total protein appeared to be due to variations in the globulin fraction (Table 5). Although there was a slight increase in albumin in both strains, these values were not significantly different from the control group within the same strain. In the commercial strain, increases in serum globulin were observed with ammonia and temperature stress alone; however, significant increases were noted in all groups compared to the control in the GIANT-G9. Overall, globulin levels were higher in the GIANT‐G9 than in the commercial strain, including in the control group without stress.

Regarding lysozyme activity, stressors significantly increased this enzyme’s activity in the commercial strain, with slight differences among stress groups (Table 5). In the GIANT-G9, lysozyme activity increased under temperature stress but markedly decreased after exposure to ammonia and both stressors. A significant interaction between strain and stress was observed for all parameters except albumin.

**Fig. 4:**
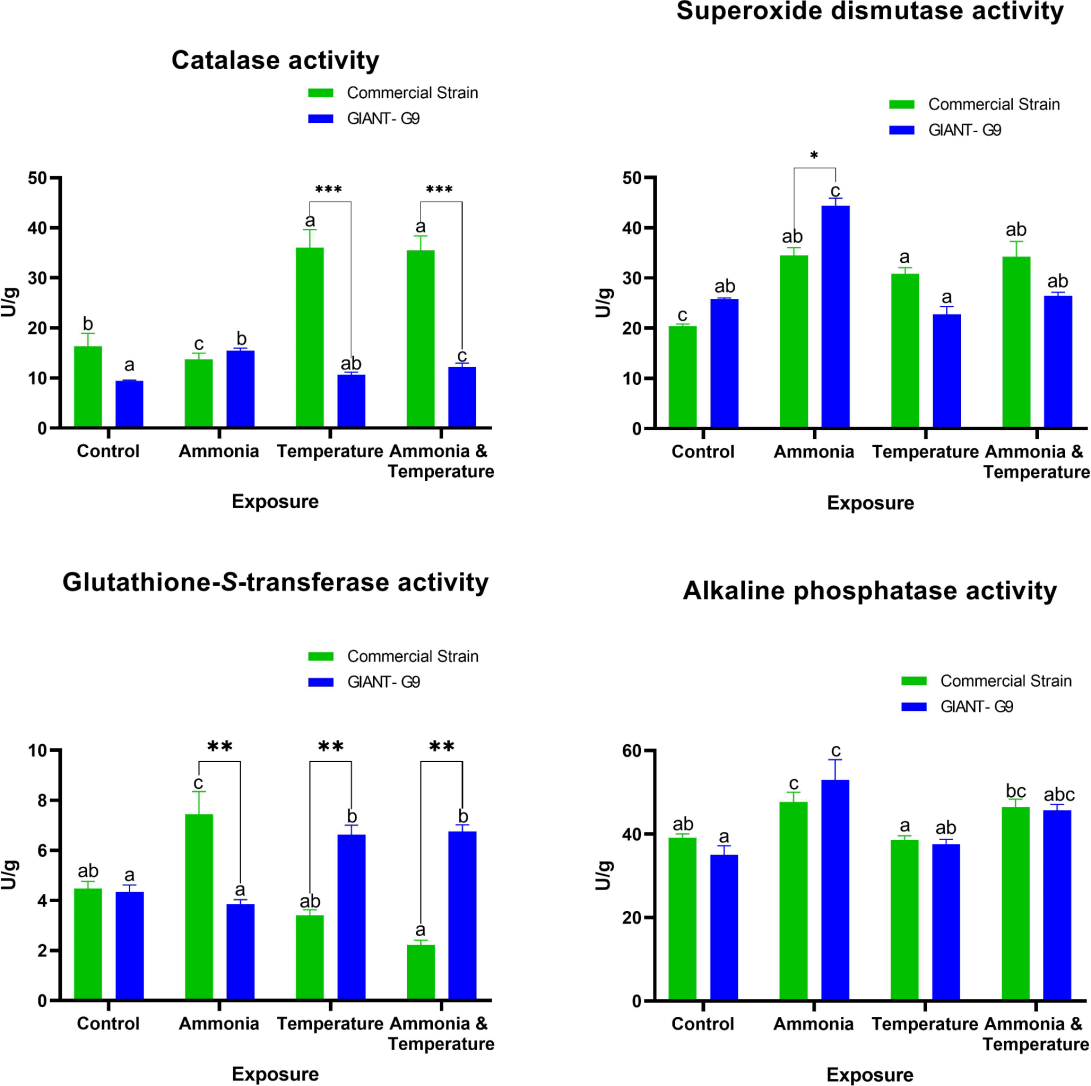
Liver enzyme activities of superoxide dismutase (SOD), catalase, glutathione-*S*-transferase (GST), and alkaline phosphatase (ALP) in two Nile tilapia strains (a commercial strain and the GIANT-G9) after exposure to control (no stress), ammonia (TAN at 10 mg/L), temperature (37ºC), and both ammonia and temperature for three days. Data are expressed as mean ± SD. Values sharing no common lowercase letters are significantly different within the same strain, while asterisks denote significance within the same stress factor across different strains

The liver catalase activity decreased after ammonia stress but markedly increased after temperature stress and exposure to both stressors. These increases were significant in the GIANT-G9 under the same stress factor (Fig. 4). Specifically, in the GIANT‐G9, liver catalase activity increased with ammonia exposure and under both stressors. In the commercial strain, SOD levels increased after exposure to all stressors compared to the control, with no significant differences among the stressors (Fig. 4). For the GIANT‐G9, a significant increase in SOD was observed only in the ammonia group, which was significantly different from the commercial strain. Liver GST activity significantly increased after ammonia exposure in the commercial strain but decreased non-significantly in other stress groups compared to the control (Fig. 4). In the GIANT‐G9, liver GST activity significantly increased with temperature stress and under both stressors. The variations between strains were significantly different under the same stress conditions. Liver ALP activity significantly increased in fish exposed to ammonia stress in both strains compared to the control, with no significant difference between the strains (Fig. 4). A significant interaction between strain and stress was observed for these enzyme activities. parameters.

### Expression of immune-related genes after exposure to stressors

**Fig. 5:**
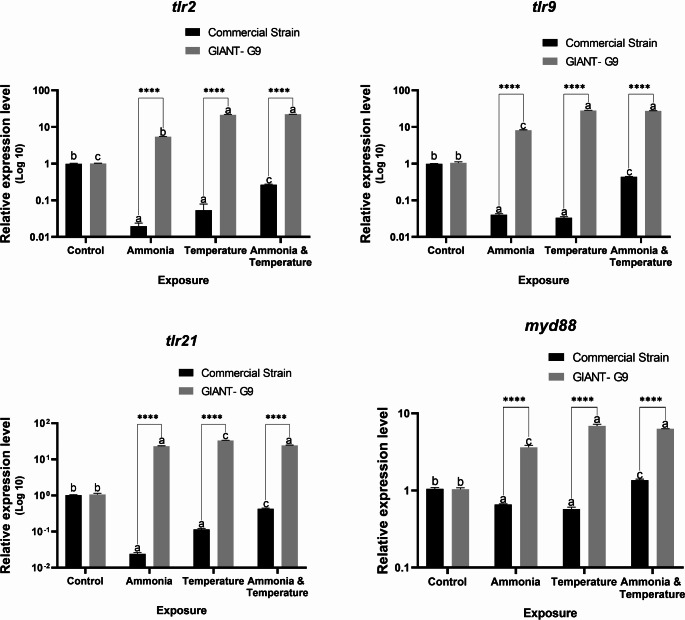
Relative expression levels of Toll-like receptors (*tlr*) 2, 9, and 21, and myeloid differentiation primary response protein 88 (*myd88*) genes in the spleen of two Nile tilapia strains (a commercial strain and the GIANT-G9) after exposure to control (no stress), ammonia (TAN at 10 mg/L), temperature (37ºC), and both ammonia and temperature for three days. The expression levels were normalized using the geometric mean of two reference genes, *ef1α* and *ubce*, following the Pfaffl (2001) method. Data are expressed as mean ± SD. Values sharing no common lowercase letters are significantly different within the same strain, while asterisks denote significance within the same stress factor across different strains

The expression of *tlr2*, *tlr9*, and *tlr21* in the spleen was markedly down-regulated in the commercial fish exposed to stress, particularly ammonia and temperature. However, these genes were markedly up-regulated in the GIANT-G9 (Fig. 5). A similar trend was observed for splenic *myd88*, except in fish exposed to both ammonia and temperature, where it was up-regulated in the commercial strain (Fig. 5).

**Fig. 6:**
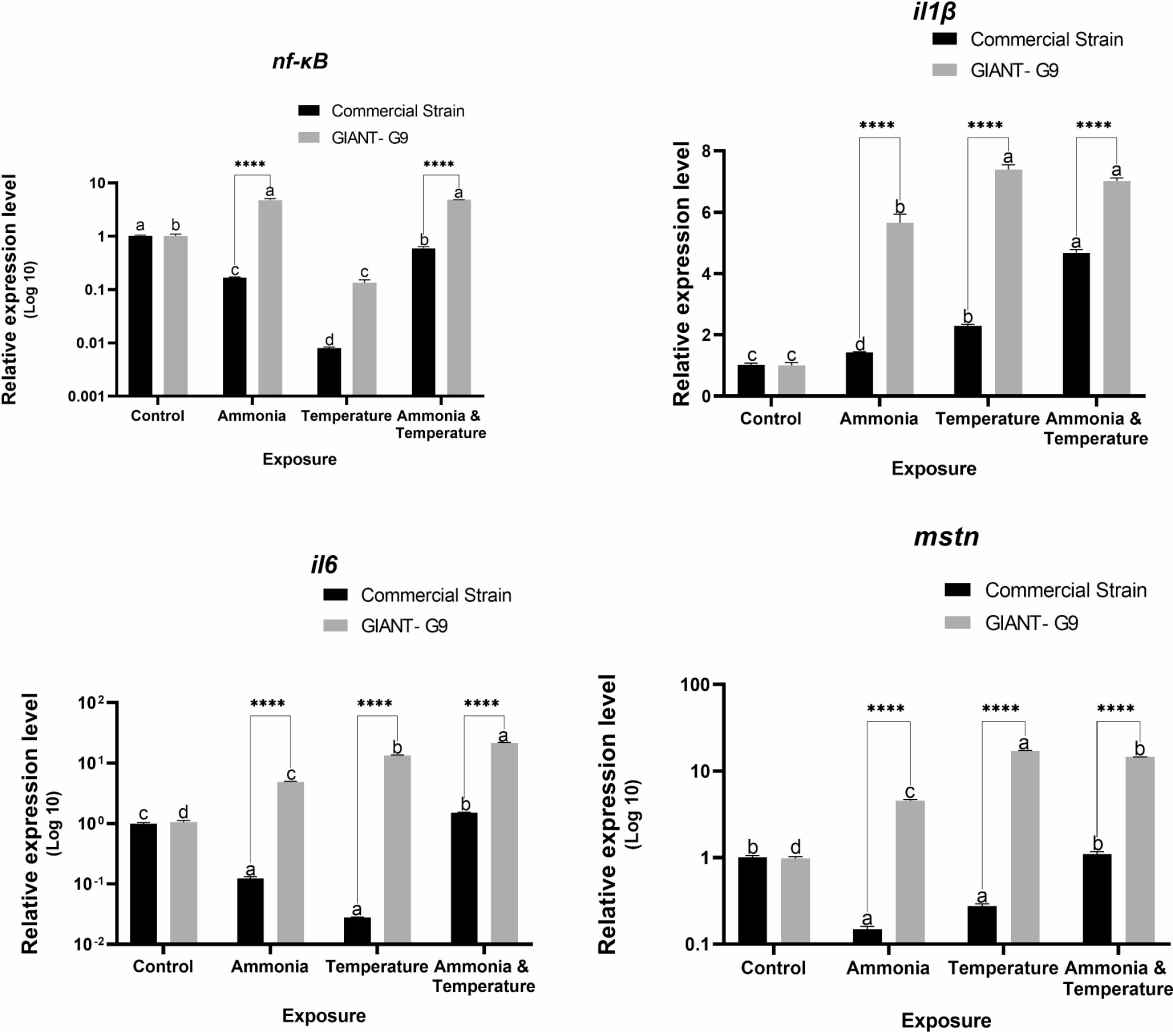
Relative expression levels of nuclear factor kappa B (*nf-κB*), interleukin (*il*) 1β and 6, and myostatin (*mstn*) genes in the spleen of two Nile tilapia strains (a commercial strain and the GIANT-G9) after exposure to control (no stress), ammonia (TAN at 10 mg/L), temperature (37ºC), and both ammonia and temperature for three days. The expression levels were normalized using the geometric mean of two reference genes, *ef1α* and *ubce*, following the Pfaffl (2001) method. Data are expressed as mean ± SD. Values sharing no common lowercase letters are significantly different within the same strain, while asterisks denote significance within the same stress factor across different strains

The splenic expression of *nf-κB* was down-regulated in the commercial strain, with particularly lower expression after temperature exposure (Fig. 6). In the GIANT-G9, the splenic expression of *nf-κB* was massively increased in fish exposed to ammonia and both stressors, while it was massively down-regulated in fish exposed to temperature alone. The splenic expression of *il1β* was up-regulated in response to stress in all groups, with massive expression in the GIANT‐G9 (Fig. 6). Similarly, splenic *il6* was up-regulated in the GIANT‐G9 (Fig. 6). However, in the commercial strain, *il6* was down-regulated in fish exposed to ammonia or temperature stress and up-regulated in those exposed to both stressors. The splenic expression of *mstn* was markedly up-regulated in the GIANT‐G9 exposed to stress and down-regulated in the commercial strain exposed to ammonia or temperature stress. Significant interaction effects between strain and stress factors were observed in gene expression in the spleen.

**Fig. 7:**
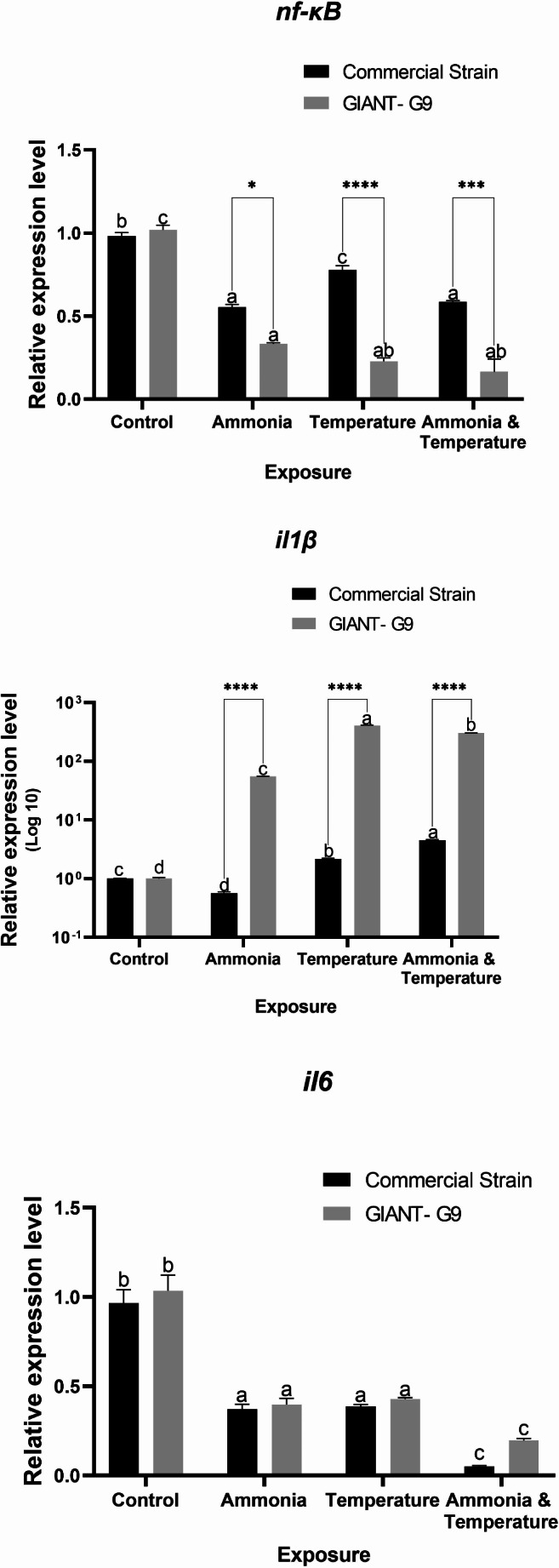
Relative expression levels of nuclear factor kappa B (*nf-κB*) and interleukin (*il*) 1β and 6 genes in the head kidney of two Nile tilapia strains (a commercial strain and the GIANT-G9) after exposure to control (no stress), ammonia (TAN at 10 mg/L), temperature (37ºC), and both ammonia and temperature for three days. The expression levels were normalized using the geometric mean of two reference genes, *ef1α* and *ubce*, following the Pfaffl (2001) method. Data are expressed as mean ± SD. Values sharing no common lowercase letters are significantly different within the same strain, while asterisks denote significance within the same stress factor across different strains

In the head kidney, the expression of *nf-κB* was down-regulated in both strains under stress, with markedly lower values in the GIANT-G9 (Fig. 7). The downregulation followed a descending trend from ammonia, temperature, to both stressors. However, in the commercial strain, fish exposed to temperature stress showed a lesser degree of downregulation. The expression of *il1β* was up-regulated in all groups under stress, except for a downregulation in the commercial strain exposed to ammonia (Fig. 7). Notably, in the head kidney, *il1β* expression showed 2- to 4-fold increases for temperature stress and both stressors, respectively, in the commercial strain, while showing near 55-, 400-, and 300-fold increases in the GIANT‐G9. The expression of *il6* in the head kidney was down-regulated similarly in both strains, except in those exposed to both stressors, where lower downregulation was observed in the commercial strain (Fig. 7). Significant interaction effects between strain and stress factors were observed in gene expression in head kidney, except for *il6* expression.

## Discussion

Achieving optimal growth performance along with balanced or enhanced health is the ultimate goal in animal production, particularly in aquaculture. The findings indicated that the GIANT-G9 demonstrated significantly better growth performance compared to a commercial strain. The brain expression of *gh* and *ghr1* increased significantly during the initial raising period but tended to decrease afterward, with *ghr1* levels remaining higher than those of the commercial strain. After 6 weeks, *gh* expression in the brain was higher in the GIANT‐G9. In contrast, the expression of *igf1* and *igf2* in the muscle changed significantly only at 12 weeks, with notable increases in the GIANT‐G9.

The growth hormone-insulin-like growth factor (Gh-Igf) system is a key modulator of growth and health in vertebrates (Fuentes et al. 2013; Gabillard et al. 2003). It has been found that *igf1ra* mRNA is most abundant in the skeletal muscle (Mohammed-Geba et al. 2016). Additionally, *igf1ra* expression increases while *gh* decreases in response to Igf1 stimulation (Mohammed-Geba et al. 2016). Fuentes et al. (2012) explored the remarkably slow growth of fine flounder and found an inherent systemic and muscle Gh resistance, with relatively high levels of plasma Gh and low plasma Igf1, as well as a higher content of truncated Ghr compared to full-length Ghr in the skeletal muscle. This was accompanied by impaired function of the Janus kinase 2 (JAK2)-signal transducers and activators of transcription 5 (STAT5) signaling pathway and decreased *igf1* expression (Fuentes et al. 2012). This study also elucidated that the interplay between *gh* and *igf1* is a key factor in regulating growth. These findings are supported by evidence from other species such as Atlantic halibut (*Hippoglossus hippoglossus*) and rainbow trout (*Oncorhynchus mykiss*) (Fuentes et al. 2013; Hildahl et al. 2008). In transgenic zebrafish that constantly overexpress full-length Ghr1, but not truncated Ghr1, there was increased expression of *igf1* and higher growth rates compared to the control (Ishtiaq Ahmed et al. 2011). Notably, in the current study, the expression of *ghr1* remained at higher levels in the GIANT-G9 compared to the commercial strain, regardless of the variations in *gh*, *igf1*, and *igf2* expression.

Although Igf1 plays essential roles in protein synthesis and muscle growth in fish (Duran et al. 2022). While Igf1, but not Igf2, synergizes and interacts with Gh, Igf2 has a stronger effect on myocyte proliferation (Rius-Francino et al. 2011). The effect of Gh on the expression of *igf1* and *igf2* is modulated by insulin and cortisol (Pierce et al. 2011). Although *igf2* does not seem to affect the body size of fish during the early stages of life (Schrader and Travis 2012), it is positively linked to growth in later stages. Notably, variations in the genetic makeup of *igf2* have been associated with significant effects on male growth in the GIFT strain (Juhua et al. 2010), as well as total length and body weight in European sea bass (*Dicentrarchus labrax*) (Ozcan-Gokcek et al. 2020), and growth traits in Spotted sea bass (*Lateolabrax maculatus*) (Fan et al. 2023) and pikeperch (*Sander lucioperca*) (Teng et al. 2020).

The GIANT-G9 appears to have more positive regulation of *gh*, *ghr1*, *igf1*, and *igf2*, leading to better growth performance across various life stages compared to the commercial strain.

The GH-IGF system plays a crucial role in regulating the body’s response to stress (Gabillard et al. 2003). Zhong et al. (2022) proposed that Igf1 modulates the expression network related to complement and coagulation cascades in tilapia, highlighting its involvement in disease defense. Additionally, changes in rearing temperature affect the muscle expression of *igf1* and *igf2* without influencing growth. However, temperature affects growth by stimulating hepatic Igf1 secretion under the influence of Gh (Gabillard et al. 2003).

In tilapia, Gh treatment has been shown to increase *igf1* mRNA levels in the head kidney more than in the liver, and to increase *igf2* mRNA in the liver, as well as *igf1* and *igf2* in the spleen, while decreasing *tnfα* expression in both the head kidney and spleen (Shved et al. 2011). A similar trend was observed in the head kidney leukocytes of Atlantic salmon, where Gh treatment down-regulated *tnfα* and *il8* while upregulating *il1β* expression (Pontigo and Vargas-Chacoff 2021). Cortisol, the primary stress hormone, and Tnfα, have been found to increase the expression of suppressors of cytokine signaling, inhibit Gh signaling via the JAK2/STAT5 pathway, and suppress *gh*-stimulated *igf1* mRNA abundance (Jiang et al. 2020; Philip and Vijayan 2015).

Antioxidant and immune systems are the first lines of defense against stress, helping to maintain normal body physiology. Toll-like receptors are among their tools, recognizing pathogen-associated molecular patterns and thereby triggering immune responses (Sundaram et al. 2012; Yao et al. 2023). These receptors also respond to environmental changes such as salinity, temperature, and dietary alterations (Schmitz et al. 2017; Yao et al. 2023; Zhang et al. 2023).

In the current study, splenic *tlr* expression was markedly up-regulated in the GIANT-G9 exposed to stress, while it was down-regulated in the stressed commercial strain. This was accompanied by upregulation of splenic myd88, *nf-κB*, *il1β*, and *il6* in the stressed GIANT‐G9. This could be linked to the function of Tlr in activating a variety of proinflammatory cytokine and chemokine production, the crucial role of Nf-κB in regulating inflammatory responses (Qiu et al. 2020), and the central role of Myd88 in the Tlr/Il1R activation cascade (Chen et al. 2020; Trung and Lee 2020). Additionally, in the GIANT‐G9, the expression of *il6* was markedly higher than *il1β* in the spleen, while the reverse was observed in the head kidney, with extensive increases in *il1β* expression, suggesting differential tissue responses to the same stressors. In the commercial strain, *il1β* expression was mildly up-regulated compared to the GIANT‐G9. In response to immune stimulation in the GIANT‐G9, globulin levels were significantly increased. However, the globulin levels in the commercial strain remained similar to those in the stressed GIANT‐G9. This difference could be explained by the upregulation of splenic *mstn* in the spleen of the stressed GIANT‐G9, as Mstn likely plays a role in immune signaling and defense capacity (Wu et al. 2020). Conversely, dietary supplementation with Mstn inhibitory peptides improved the immune capacity of sea bass (*Lateolabrax maculatus*) (Yating et al. 2017).

Interestingly, the downregulation or upregulation of most genes followed a descending or ascending trend according to the fish strain, from ammonia, temperature, to both stressors. Based on this, the question is: if the level of stress increases, does the expression of immune-related genes also increase? This could be correct if we consider that excessive immune response, particularly cytokine production, is an indicator of immune dysregulation and could hinder the physiological state of the organism (Jarczak and Nierhaus 2022). It has been found that up-regulated expression of immune genes is not necessarily associated with enhanced defense and readiness (Abo-Al-Ela 2019; Abo-Al-Ela et al. 2017). Similarly, in GIFT strain, exposure to polystyrene microplastics caused increased levels of Il1β and Tnfα in the brain and gills. This overproduction was accompanied by oxidative stress, indicating that an excessive immune response disrupts overall fish health (Zheng et al. 2023). Additionally, overactivation of Toll-like receptors causes excessive triggering of various cytokine productions, leading to detrimental inflammatory responses (Huang et al. 2020). Further evidence supports these findings, showing that suppressive proteins, such as suppressors of cytokine signaling, help mitigate and control excessive immune responses to curb harmful inflammation and tissue damage that may result from an overactive immune response (Hao and Sun 2016; Huang et al. 2020).

Exposure to ammonia and/or elevated temperature stimulated the antioxidant response, with the commercial strain demonstrating the highest activities of liver catalase and SOD. Additionally, serum lysozyme activity increased in the commercial strain under stress. Under temperature stress, lysozyme activity also increased, whereas it decreased under other stress conditions in the GIANT-G9. The individual variations between the two strains in modulating antioxidant and immune systems in response to stress indicate a more functional response and lower stress levels in the commercial strain compared to the GIANT‐G9.

The increased immune stimulation, combined with higher ROS levels, may have hindered the physiological status, resulting in higher mortality in the GIANT-G9 compared to the commercial strain. Rapid growth rates may reduce the energy and nutrients available for normal immune and antioxidant functions, leading to some degree of immune suppression. Fish that are rapidly growing or in specific physiological states have high metabolic activity, which elevates ROS production (Birnie-Gauvin et al. 2017; Mortelette et al. 2010). This was also observed through greater liver injury, indicated by higher serum levels of ALT, AST, and ALP.

Exposure to stress triggers various physiological processes that activate the body’s antioxidant defense and immune system to maintain balance. However, this can sometimes lead to oxidative stress and system failure. Therefore, ensuring a balanced response and functional body systems is crucial for preserving organism performance and life.

Selective breeding is a well-established tool with a substantial impact on the aquaculture sector and will continue to drive its growth in the present and future (Cavatti Neto et al. 2023; Janssen et al. 2017). Worldwide, researchers and various countries are working to develop and implement tilapia strains with high growth performance at minimal cost (Basiao et al. 2005; Cavatti Neto et al. 2023; Janssen et al. 2017). These efforts focus on enhancing physiological traits and stress resistance in strains, including tolerance to temperature extremes, low oxygen levels, and disease resistance. Additionally, adaptability traits, which enable fish to better cope with environmental changes, are a key consideration (Hu et al. 2024).

Whole-genome selective breeding, or genomic selection, is a powerful tool that can help identify strains with more balanced characteristics, such as improved stress resistance, while also shortening the breeding cycle (Hu et al. 2024; Yáñez et al. 2023). For instance, genome-wide association studies and genomic prediction have successfully identified quantitative trait loci (QTLs) for acute hypoxia tolerance in rainbow trout (Prchal et al. 2023).

Selective breeding has also produced favorable outcomes in population resilience, as shown by coral strains with improved heat tolerance (Humanes et al. 2024), enhancing genomic selection rates among candidates. However, selective breeding may unintentionally overlook traits like temperature tolerance. For example, Rezk and Kamel (2011), reported variability in cold tolerance selection effects between *O. niloticus* and *O. aureus*, highlighting the importance of considering a comprehensive set of traits in breeding programs.

Similarly, studies on red tilapia, Genomar Supreme Tilapia (GST; GIFT-derived and developed further in the Philippines), and GIFT strains revealed comparable growth patterns when reared at 22 °C. However, at 30 °C, GIFT and GST exhibited superior growth compared to red tilapia (Santos et al. 2013). These findings underscore the role of temperature as a critical modulator of fish growth, even among different strains. Moreover, under high stocking densities, improved strains such as the sixth generation of GIFT and the 13th-generation FaST selected line (Freshwater Aquaculture Center Selected Tilapia) showed significantly higher growth compared to non-improved commercial strains (Ridha 2006).

Environmental conditions, such as freshwater versus brackish water, also influence growth performance. For instance, the GIFT strain demonstrated superior average weight gain compared to four other improved tilapia strains: BIG NIN (sourced from Asia), Chitralada (sourced from Asia), “Ruvu Farm” (sourced from Uganda, East Africa), and Silver YY (sourced from Europe) (Moses et al. 2021). Additionally, the GIFT strain exhibited excellent growth performance, and the genotype-by-environment interaction was minimal and not significant enough to warrant prioritization in breeding programs (Moses et al. 2021).

Under various rearing conditions, the specific growth rate of the GIFT strain ranked highest, followed by FaST, control stock NIFI (National Inland Fisheries Institute, Thailand), and SEAFDEC-selected stocks (Southeast Asian Fisheries Development Center, Philippines). These strains were evaluated under both controlled tank-rearing (farm conditions) and lake-based cage systems (uncontrolled conditions) for Nile tilapia, with no significant differences in performance between the two rearing environments (Romana-Eguia et al. 2010). The variation in growth observed among the Nile tilapia stocks, particularly under controlled tank farming conditions, was primarily attributed to genetic factors, specifically differences between stocks (Romana-Eguia et al. 2010).

GIANT, a tilapia strain developed by WorldFish in Egypt, has demonstrated excellent growth performance and net profits compared to commercial strains (Ibrahim et al. 2019). Since its establishment in 2002, the Abbassa strain has maintained high levels of genetic diversity (Nayfa et al. 2020), indicating a robust genetic structure. However, in its juvenile stages, the GIANT-G9 appeared to be more susceptible to stress. Maintaining a balanced physiological response throughout an organism’s lifespan can be complex, but future research on different life stages of the GIANT‐G9 should provide more insight into its stress tolerance capabilities. Including larger experimental populations will also offer a clearer understanding. Generally, it is recommended to consider strain improvements based on selecting organisms that are more immune and stress-resistant, in addition to achieving the highest growth rates.

## Conclusion

Selective breeding is a powerful, long-term, and labor-intensive tool for developing aquaculture stocks with desirable characteristics. The GIANT-G9 exhibited better growth performance compared to the commercial strain. However, the commercial strain demonstrated a more balanced and functional antioxidative response to stress at the certain stage of the rearing cycle. It is highly recommended to conduct more research on different stages of the production cycle and to focus on maintaining relatively balanced characteristics to develop a stronger strain.

## Data Availability

The majority of the data supporting the findings of this study are included within the manuscript. Other relevant datasets can be obtained from the corresponding author upon reasonable request.
